# The Frontier of Melanoma Treatment: Defeating Immunotherapy Resistance—A Systematic Review

**DOI:** 10.32604/or.2025.070505

**Published:** 2026-01-19

**Authors:** Kamila Mozga, Olga Synowiecka, Igor Rydzyk, Anna Marek, Ewelina Wieczorek, Alicja Petniak, Paulina Gil-Kulik

**Affiliations:** 1 Student Scientific Society of Clinical Genetics, Medical University of Lublin, Lublin, 20-080, Poland; 2 Department of Clinical Genetics, Medical University in Lublin, Lublin, 20-080, Poland

**Keywords:** Melanoma, immunotherapy, immune checkpoint blockade (ICB), immunoresistance, systematic review

## Abstract

**Objectives:**

Immunotherapy based on immune checkpoint blockade (ICB) has become a key treatment for melanoma. However, the increasing number of cases of melanoma resistant to immunotherapy highlights the need to develop methods to overcome this resistance. This study aims to collect the most recent information on melanoma immunotherapy, discuss potential strategies to overcome resistance to immunotherapy, and identify areas that require further analysis.

**Methods:**

To achieve this goal, scientific publications from 2021–2024 available in PubMed and Google Scholar databases were analyzed. The databases were searched using the following terms: “melanoma”, “immunotherapy”, “Immune Checkpoint Blockade”, and “immunoresistance”.

**Results:**

The results of preclinical and early-stage clinical research indicate the potential application of tank-binding kinase 1 (TBK-1), fecal microbiota transplant (FMT), Toll-like Receptor 9 (TLR9), lipid nanoparticles (LNPs) containing a stimulator of an interferon gene agonist (STING), BRAF inhibitors, Lymphocyte Activation Gene (LAG-3), T-Cell Immunoglobulin and ITIM Domain (TIGIT), and oncolytic viruses (OVs) as potential methods to enhance melanoma sensitivity to ICB.

**Discussion:**

To optimize immunotherapy, further research is needed to determine the detailed mechanisms of action, safety profiles, tolerability, and precise patient selection criteria for methods capable of overcoming melanoma’s immunoresistance

## 1 Introduction

Melanoma is one of the most aggressive human neoplastic tumors [1,2]. Its high aggressiveness is rooted in its rapid growth and high capability of invasion into deeper layers of the skin. Melanomas can metastasize, even in the early stages of development. Metastasis is a poor prognostic indicator [1]. The incidence of melanoma is increasing from year to year [1,2]. According to statistics from the National Cancer Institute in the USA, in terms of frequency among malignant tumors, melanoma was ranked 15th in 2021 and fifth in 2022 [3]. In Europe, countries such as Denmark, the Netherlands, and Sweden have the highest occurrence of melanoma [4].

Melanoma originates from melanocytes, which are derived from the neural crest of the ectoderm [1]. Melanoma can develop from pre-existing benign or *de novo* lesions [3]. Risk factors for developing melanoma include unmodifiable factors such as age, sex, race, and genetic predisposition, and modifiable factors such as environmental conditions, the number of nevi, and exposure to ultraviolet (UV) radiation [2].

The World Health Organization (WHO) classifies melanoma based on location (skin or mucous membranes), cell type, histology, and UV radiation exposure [4]. UV-dependent types of melanoma include superficial spreading melanoma, lentigo malignant melanoma, and desmoplastic melanoma [4]. On the other hand, there are also melanocytic tumors not associated with UV exposure. Uveal, acral lentiginous, mucosal, and blue nevus melanomas are classified as non-UV-dependent [4]. Nodular melanoma represents a particular form of tumor, likely the final stage of the previously mentioned types, and has the worst prognosis [4].

Common therapeutic methods for melanoma include surgical excision, chemotherapy, radiotherapy, immunotherapy using checkpoint inhibitors, targeted therapy, and regional therapies [2,5]. The melanoma stage is the deciding factor in the choice of a particular method [5]. In cases of advanced-stage disease, there is a need for systemic therapy because surgical treatment alone is insufficient [5]. Currently, the therapy selection is highly individualized; however, chemotherapy has been the first choice for many years, but this is no longer the primary treatment option [2]. The identification of mutations in melanoma has paved the way for the development of targeted therapies [1,2]. The greatest advantage of targeted therapy is better patient outcomes than those of traditional chemotherapy. However, the main drawback is the development of resistance in cancer cells to treatment during therapy [1,2,6].

The aim of our study was to gather the latest information on melanoma immunotherapy, discuss its associated benefits and future perspectives, and identify areas that require further analysis.

### 1.1 Melanoma

From a histological perspective, the most common cells found in melanomas are epithelioid or spindle cells [1]. Often, a tumor may contain both cell types, leading to its classification as “mixed” melanoma [4].

Lymphatic infiltration is a characteristic feature of the tumor microenvironment (TME) [1]. Consequently, spontaneous regression in the primary focus sometimes occurs, indicating an anti-tumor response from the body’s immune system [2]. Studies have shown that the total count of infiltrating immune cells in skin melanoma metastases correlates with patient survival [3].

The gene most frequently implicated in the pathogenesis of this tumor is V-Raf Murine Sarcoma Viral Oncogene Homolog B (*BRAF*), specifically the *BRAF* V600E variant [3]. This mutation activates the mitogen-activated protein kinase (MEK) pathway, leading to uncontrolled and abnormal cell proliferation [3]. The second most commonly detected gene is neuroblastoma RAS viral oncogene homolog (*NRAS*). The RAS family also activates the MEK pathway and increases the resistance to *BRAF* inhibitor therapy [3]. Neurofibromin 1 (*NF1*) gene loss has been discovered in one-third of patients with melanoma, leading to RAS activation [3]. Other genes involved in melanoma pathogenesis include phosphatase and tensin homolog (*PTEN*), *KIT* proto-oncogene, receptor tyrosine kinase (*c-KIT*), melanocyte inducing transcription factor (*MITF*) cyclin dependent kinase inhibitor 2A (*CDKN2A*), cyclin dependent kinase 4 (*CDK4*), and BRCA1 associated deubiquitinase 1 (*BAP1*) [3]. Melanoma cells often exhibit mutations in chromosomal regions in which genes encoding human leukocyte antigen (HLA) class I heavy chains are located, favoring immune resistance owing to altered antigen presentation to cytotoxic CD8+ T lymphocytes [3,7].

Classical morphological analysis of cells is not the only way to diagnose and confirm the melanocytic origin of a tumor [3]. Modern genetic techniques, including immunohistochemical (IHC) markers, are available [3]. Noteworthy markers for diagnosing melanoma include SOX10, S100, melanoma antigen recognized by T cells 1 (Melan A), and human melanoma black (HMB45) [1]. One of the latest discoveries is the immunomarker preferentially expressed antigen in melanoma (PRAME), valued for its high specificity in detecting metastases [1,7]. Also recently described high-resolution proton magnetic resonance spectroscopy as a noninvasive method for assessing *in vivo* predictive biomarkers of metabolic response in melanoma patients treated with signaling-inhibiting therapies [8].

### 1.2 Immunotherapy in Melanoma

Melanoma was one of the first cancers to be treated with immunotherapy [9]. Immunotherapy for melanoma is based on the use of immunomodulatory agents [9] and ICB [10].

Immunomodulatory agents include interleukin-2 (IL-2) and interferon-alpha (IFN-α) [9]. However, the use of IL-2 is heavily restricted [11]. In patients receiving high doses of IL-2, symptoms such as fever, hypotension, pulmonary edema, cardiac rhythm disturbances, and shock were observed [11]. Similarly, IFN-α monotherapy does not yield the expected results because of a low response and accumulating toxic dose [6,9].

ICB therapy involves the use of antibodies targeting programmed cell death protein 1 (PD-1), programmed death-ligand 1 (PD-L1), and cytotoxic T-lymphocyte-associated protein 4 (CTLA-4) [12].

#### 1.2.1 Anti-CTLA-4 Therapy

CTLA-4 primarily plays a modulatory role in stimulating T lymphocytes in lymph nodes by inhibiting the activation of T lymphocytes and halting the production of effector T lymphocytes [13]. The monoclonal antibody approved by the Food and Drug Administration (FDA) in 2011 for this purpose is ipilimumab [1,14].

#### 1.2.2 Anti-PD-1 and Anti-PD-L1 Therapy

PD-1 is expressed more broadly than CTLA-4 and can be found in T cells, B cells, and natural killer (NK) cells [1]. The PD-L1 has wide expression in cells of the immune system, including T cells, B cells, dendritic cells, and macrophages [14]. Antibodies used in therapies targeting PD-1 and PD-L1 include nivolumab, pembrolizumab, and atezolizumab [5]. In melanoma, anti-PD-1 therapy is believed to induce tumor rejection by reactivating CD8+ T lymphocytes [13].

#### 1.2.3 Combined Immunotherapy

Both anti-CTLA-4 and anti-PD-1/anti-PD-L1 antibodies are employed independently to treat advanced metastatic melanoma and are also utilized as adjuvant therapy for post-resection disease [13]. A combination of ipilimumab and nivolumab has been used to treat metastatic melanoma [13]. Comparing patients treated with the combination therapy of nivolumab and ipilimumab to those treated with these drugs in monotherapy, an increase in the median overall survival was demonstrated in patients receiving the combined therapy [13]. Unfortunately, combination therapy has a higher incidence of adverse effects [13].

#### 1.2.4 Side Effects

Enhancing the immune response through immunotherapy has both local and systemic implications, posing the risk of immune-related adverse events (irAE) [13]. In combination therapies, a higher incidence of irAE was observed compared to monotherapy targeting immune checkpoints [13]. The most common symptoms include skin manifestations, such as psoriasis, bullous pemphigoid, erythematous lupus, and acquired vitiligo [13]. The systemic symptoms include intestinal inflammation, hepatitis, pneumonitis, and endocrinopathy. Steroids have been used to treat irAE [13].

#### 1.2.5 Treatment Choice

In 2023, the Society for Immunotherapy of Cancer (STIC) published clinical practice guidelines for immunotherapy in melanoma treatment [13]. Experts emphasize that immunotherapy should be individually tailored for each patient, considering the stage of melanoma, the patient’s age, and coexisting conditions [13]. The assessment of PD-L1 expression has been noted by experts to be associated with the response to ICB therapy in melanoma; however, it should not be used to make clinical decisions due to inconsistencies in results regarding predictive and prognostic value [13].

Experts recommend the use of combination therapy with ipilimumab and nivolumab for stage IV melanoma in patients with poor prognostic features such as liver metastases, brain metastases, *BRAF* mutations, or high lactate dehydrogenase (LDH) levels [11]. However, for patients with a lower likelihood of tolerating irAE of high severity or for those with desmoplastic melanoma, anti-PD-1 monotherapy is preferred [11].

#### 1.2.6 TME

Characterization of the TME serves as a potential biomarker for the response to ICB in metastatic melanoma [15]. The TME consists of cancer cells, immune system cells, stromal cells, blood vessels, and other mesenchymal cells [15]. Recent reports have confirmed that chronic inflammation and IL-6 cytokine signaling are implicated in resistance to immunotherapy [12]. Inhibiting these cytokines in preclinical mouse models in combination with ICB has been shown to improve immunotherapy outcomes [12]. Studies have also shown that intratumoral CD16+ macrophages are associated with treatment outcomes in patients with metastatic melanoma treated with a combination of anti-PD-1 and anti-CTLA-4 therapies [15].

## 2 Material and Methods

### 2.1 Protocol

In our review, we used the Preferred Reporting Items for Systematic Reviews and Meta-Analyses 2020 (PRISMA 2020) guidelines [16]. The study protocol was not registered. In our review, we used the Preferred Reporting Items for Systematic Reviews and Meta-Analyses 2020 (PRISMA 2020) guidelines (PRISMA checklist provided as Supplementary Material).

All items included in the PRISMA checklist are covered in the respective subsections of the article.

### 2.2 Data Sources and Search Strategy

A comprehensive electronic search was performed by the reviewers across multiple databases—PubMed (MEDLINE), Google Scholar, and ClinicalTrials. gov.—for studies published from 01 January 2021 to 23 May 2024.

The population included patients with melanoma, as well as animal models and cell lines. All selected studies investigated cases in which ICB therapy demonstrated a limited or unsatisfactory response. Comparisons were drawn between treatment responses and the potential adverse effects of combination strategies. The primary outcome of interest was diagnostic and therapeutic utility.

Exclusion criteria included conference abstracts without full text, narrative reviews, and articles not reporting primary or systematic data relevant to interventions. The search strategy combined MeSH terms and the following keywords: “melanoma”, “immunotherapy”, “Immune Checkpoint Blockade”, and “immunoresistance”.

### 2.3 Eligibility Criteria

Eligible studies evaluated treatment response rates, treatment-related adverse events, toxicities, clinical benefits, survival outcomes, or identified combination strategies that enhanced responses to ICB in melanoma.

Only studies published in English or Polish were included to ensure accuracy and minimize potential translation errors.

The inclusion criteria comprised studies investigating combination therapies with ICB in melanoma, using cell lines, animal models, or patients. Studies conducted exclusively on non-melanoma material were excluded.

Due to the heterogeneity of the included publications, the analyses were stratified into eight subgroups based on the type of combination therapy evaluated.

### 2.4 Data Collection and Selection Process

In the first stage of the selection process, duplicate records were removed using a free online software, followed by manual verification. Studies addressing the main concept were identified by screening titles, abstracts, and keywords. Articles written in languages other than English or Polish were excluded.

To ensure that only articles aligned with the study’s aims and inclusion criteria were included, a second screening stage was conducted. The abstracts of the remaining sources were reviewed to assess their relevance and to determine whether they addressed combination therapy involving ICB.

The selection of articles was conducted by three reviewers. Two reviewers independently performed the initial screening, after which the selected articles were cross-checked by an additional reviewer. No inconsistencies were identified between reviewers during the selection process.

### 2.5 Data Items and Effect Measures

From the selected studies, the following data were extracted: study type (clinical or preclinical), experimental system (human subjects, animal models, or cell lines), and therapeutic strategy investigated. The approaches were categorized into four main groups: (1) modulation of innate immune signaling, (2) microbiome-based interventions, (3) nanoparticle-mediated delivery of immune stimulators, and (4) targeted therapies.

Effect measures were reported according to the intervention and study setting. These included tumor growth and regression in preclinical models, patient response rates in clinical studies, and immunological endpoints such as T-cell infiltration, interferon signaling, and cytokine expression. When available, safety profiles, tolerability, and treatment-related adverse events were also recorded.

### 2.6 Study Risk of Bias Assessment

All randomized controlled trials (RCTs) were assessed for risk of bias using the Cochrane Risk of Bias 2.0 tool (RoB 2.0) [17]. Non-randomized studies were evaluated using the ROBINS-I tool (Risk of Bias in Non-randomized Studies—of Interventions) according to Cochrane recommendations. Both assessments were performed independently by two reviewers and cross-checked for consistency. Detailed ROBINS-I and RoB 2.0 evaluations are available upon request.

### 2.7 Study Selection

**Figure 1:**
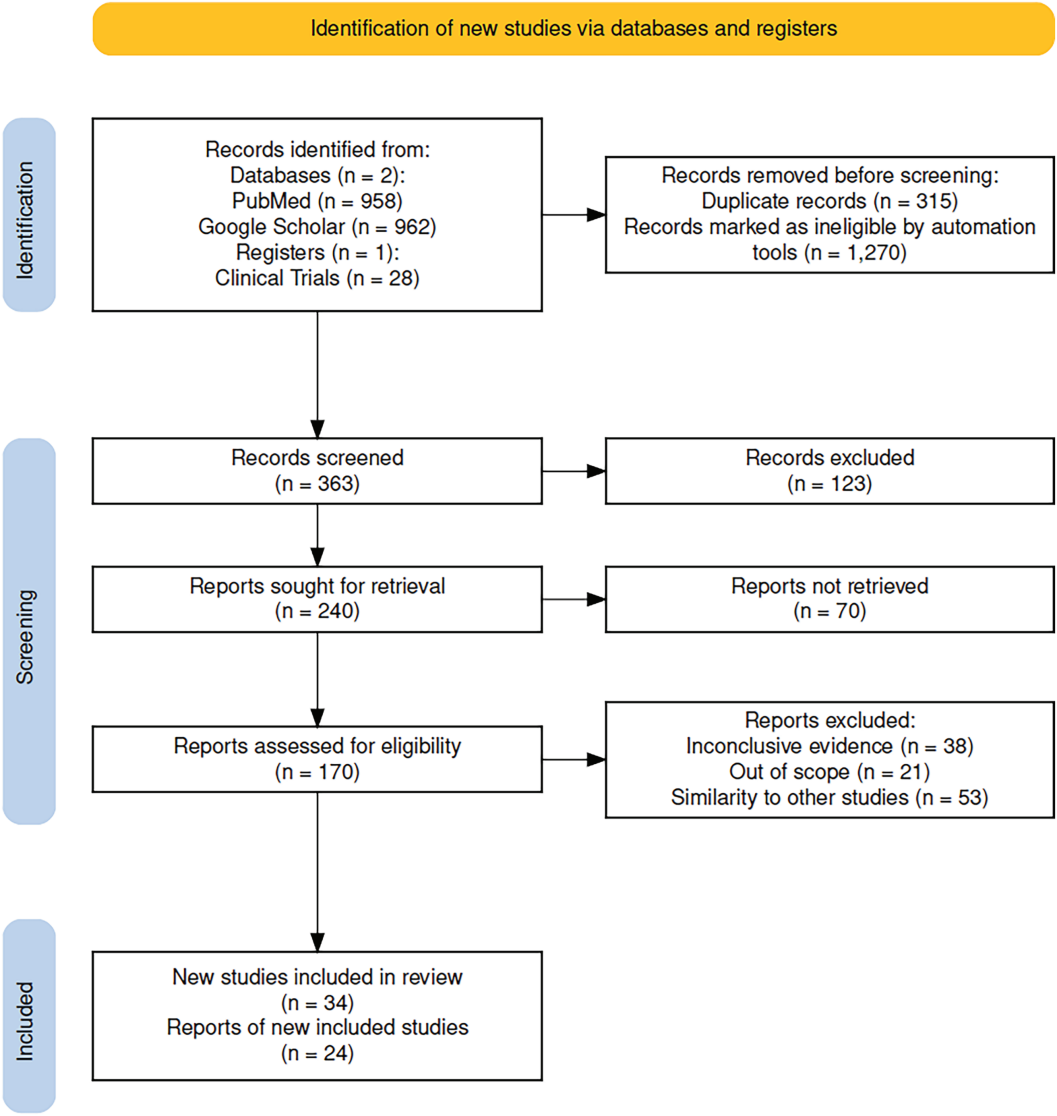
PRISMA2020 flow diagram of evaluated studies

Initially, we identified 1920 records from databases and 28 from clinical trials registries. A total of 315 records were removed as duplicates, and 1270 records were marked as ineligible by automation tools. This left 363 records to be screened. Among these, 240 reports were sought for full-text retrieval, and 170 were successfully assessed for eligibility. We excluded 112 records for reasons such as inconclusive evidence, lack of relevance to the scope of this review, or similarity to other studies. Only studies with accessible full text were included to allow data extraction; this criterion did not affect study eligibility or introduce bias, as accessibility was independent of study outcomes. Finally, 34 studies and 24 reports of new studies were included in the review (Fig. 1).

## 3 Results

In the following section authors will list and elaborate on potential strategies to overcome drug resistance in melanoma. Comparative analysis will be given in the “Discussion and Conclusions” section.

### 3.1 BRAF Inhibitor

Mutations in the *BRAF* oncogene, specifically at exon 15/codon 600, represent the most prevalent genetic changes observed in cutaneous melanoma, occurring in approximately 50% of cases [3,18]. This subtype of melanoma, characterized by *BRAF* mutations, is essential for the molecular categorization of melanoma and is classified as one of its four main types [3,18]. Melanomas with *BRAF* mutations may exhibit amplification of the PD-L1 gene, which can lead to immune resistance while simultaneously enhancing responsiveness to PD-1/PD-L1 ICBs [18]. The primary oncogenic drivers of cutaneous melanoma include mutations in *BRAF*, *NRAS*, and *c-KIT* [19]. The presence of a *BRAF* V600E mutation enables the application of targeted therapies, either as monotherapy or in combination with MEK inhibitors [19].

The presence of immunosuppressive cytokines—including IL-6, IL-8, IL-10, and chemokine ligand 2 (CCL2)—along with reduced expression of melanoma-associated differentiation antigens (such as melan-A/melanoma antigen recognized by T-cells 1 (MART-1), glycoprotein 100 (gp100), and tyrosinase) and major histocompatibility complex (MHC) class I molecules, contributes to an “immunologically cold” tumor phenotype [20]. This immunosuppressive profile limits the ability of the immune system to effectively recognize and respond to tumor cells [20]. Additionally, the TME is often dominated by immunosuppressive cell populations, including regulatory T cells and myeloid-derived suppressor cells, which are present at higher frequencies than active CD4+ and CD8+ tumor-infiltrating lymphocytes (TIL) [20].

The 2022 European interdisciplinary guidelines for melanoma recommended the administration of *BRAF* inhibitors as per specified recommendations [21]. For cases classified as distant metastases (stage IV), systemic treatment is indicated as a standard practice, regardless of whether metastases have been surgically removed or not [21]. For patients lacking *BRAF* mutations, the initial approach consists of immunotherapy using anti-PD-1 in monotherapy or in combination therapy with the addition of CTLA-4 inhibitors [21]. On the other hand, in cases of stage IV melanoma characterized by a *BRAF*-V600 E/K mutation, employing *BRAF*/MEK inhibitors serves as an alternative to immunotherapy during first-line therapy [21]. In situations where primary resistance to immunotherapy manifests among patients with a *BRAF*-V600 E/K mutation, these inhibitors are recommended as second-line therapeutic agents [21].

Triplet therapy combines the use of *BRAF* and MEK inhibitors with the addition of ICB [21]. It has demonstrated enhancements in both progression-free survival (PFS) and longevity of responses when compared to treatment using *BRAF*/MEK inhibition alone [21]. The clinical implementation of this combination, particularly in first-line metastatic cases, is currently being studied [21]. There are ongoing discussions regarding its most effective use among advancements in front-line immunotherapy [21].

Prominent combinations of *BRAF*/MEK inhibitors, including dabrafenib/trametinib, vemurafenib/cobimetinib, and encorafenib/binimetinib, have shown high objective response rates (ORR) approaching 70% among patients with advanced *BRAF*-mutant melanoma [22]. Nevertheless, resistance to these targeted therapies frequently arises, leading to a relatively brief median PFS ranging from 10 to 15 months and overall five-year survival rates between 34% and 37% [22]. In contrast, individuals within the subgroup possessing *BRAF* mutations have shown a significantly higher five-year overall survival rate of around 60% [22].

Integration of *BRAF* and MEK inhibitors with ICB during initial treatment has resulted in improvements in PFS and response durability in clinical trials [23]. The outcomes were similar to enhancements observed with targeted therapies alone [23]. The widespread use of triplet therapies in the first-line treatment of metastatic melanoma has been limited, in part, due to the lack of extensive comparisons with single-agent anti-PD-1 therapy or the combination of ipilimumab and nivolumab, both of which are becoming standard of care for patients with either *BRAF*-mutant or wild-type melanoma [23]. Ongoing research is focused on determining their optimal use across various settings, including (neo) adjuvant contexts alongside sequential and alternating therapeutic strategies [23] (Table 1).

**Table 1:** Summary of clinical trials evaluating the effects of *BRAF*/MEK inhibitors. Data were obtained from findings from www.clinicaltrials.gov using search terms “melanoma”, “*BRAF*”, and “PD-1”. Status of medical trials as of 27 September 2025

Treatment	Status	Enrolled patients	Primary outcome measures	NCT Number/Reference
Pembrolizumab, encorafenib, binimetinib	Phase 2, Completed	1	Decrease in nuclear c-MYC levels *in situ* by 50%, increase in mitochondrial proteins ACAT1 and HADHA in the tumor cells *in situ* by 50%, increase in CD8+ counts *in situ* by 50%, BORR^1^ to pembrolizumab [24]	NCT05304546
Encorafenib, binimetinib, pembrolizumab, pembrolizumab alone	Phase 1, Phase 2, Completed	16	Phase 1: Incidence of TRAE^2^ Phase 2: PFS^3^ at 12, 18, and 24 months [25]	NCT02902042
Dabrafenib, trametinib, hydroxychloroquine	Phase 1, Phase 2, Unknown Status	63*	Phase 1: Incidence of: AE^4^ Phase 2: ORR^5^ [26]	NCT03754179
*BRAF* inhibitors, MEK inhibitors, and anti-PD-1	Observational Unknown Status	18*	Comparison between intra- and extracerebral metastasis [27]	NCT05510466
*BRAF* inhibitors after immunotherapy, monotherapy, combination therapy, targeted therapy combination therapy	Observational Completed	1961	Mean age, number of male patients, race, mean CCI^6^ score, ECOG PS^7^, number of patients with study-related biomarkers, number of metastatic sites [28]	NCT05984615
Trametinib after anti-PD- and CTLA-4-blocking	Phase 2, Completed	45	ORR^5^ on trametinib and dabrafenib [29]	NCT04059224
Vemurafenib, cobimetinib, atezolizumab	Phase 2, Completed	186	Time to first documented disease progression [30]	NCT02902029
Dabrafenib, trametinib, pembrolizumab	Phase 2, Unknown Status	60	PRR^8^ [31]	NCT02858921
Binimetinib, nivolumab, questionnaire administration	Phase 2, Terminated	3	ORR^5^ [32]	NCT04375527
Encorafenib, binimetinib, pembrolizumab, ipilimumab, nivolumab	Phase 2, Active, Not Recruiting	38	ORR^5^, CR^9^, PR^10^ [33]	NCT05926960
Pembrolizumab, dabrafenib, trametinib, placebo	Phase 1, Phase 2, Completed	184	Number of participants who experienced DLTs^10^, ORR^5^, PFS3, number of participants who experienced an AE^4^, number of participants who discontinued study treatment due to an AE^4^ [34]	NCT02130466
Spartalizumab, placebo, dabrafenib, trametinib	Phase 3, Terminated	568	Number of participants with dose DLTs^11^, change from baseline in PD-L1 expression upon treatment with spartalizumab in combination with dabrafenib and trametinib, change from baseline in CD8+ cells upon treatment with spartalizumab in combination with dabrafenib and trametinib, PFS^3^ [35]	NCT02967692
Atezolizumab, cobimetinib, vemurafenib	Phase 1, Completed	67	Percentage of participants with DLTs^11^, percentage of participants with AE^4^ [36]	NCT01656642
Cobimetinib, vemurafenib, ipilimubab, nivolumab	Phase 2, Unknown Status	200*	BORR^1^ [37]	NCT02968303

Note: ^1^Best Overall Response Rate per RECIST v1.1; ^2^Treatment-related Adverse Event; ^3^Progression-free Survival per RECIST v1.1; ^4^Adverse Events; ^5^Overall Response Rate per RECIST v1.1; ^6^Charlson Comorbidity Index; ^7^Eastern Cooperative Oncology Group Performance Status; ^8^Pathological Response Rate; ^9^Complete Response; ^10^Partial Response; ^11^Dose-Limiting Toxicities; *Estimated.

### 3.2 FMT

Gut microbiota (GM) refers to the collective living microorganisms in the human digestive system [38]. This microbial population provides several benefits to the host, including supporting the integrity of the mucosal barrier, metabolizing medications and nutrients, regulating the host’s immunity, and protecting against pathogens [38].

Recent findings suggest that GM affects the response to immunotherapy, making its modulation an appealing approach for overcoming resistance to immunotherapy. There are different methods to modulate GM, such as dietary changes, but the most established method involves FMT [38]. During FMT, the donor microbiome is transferred to the recipient through endoscopic administration or capsules containing fecal suspension [38]. The effectiveness of FMT in treating *Clostridium difficile* infections has led to research exploring its potential use for various other indications. This includes investigating its efficacy in melanoma patients in the context of the ICB response [38,39].

The impact of FMT on overcoming resistance to immunotherapy was examined in patients with advanced melanoma [40]. Fifteen patients with anti-PD-1 therapy-resistant melanoma underwent FMT using donor feces from individuals who showed a positive response to pembrolizumab [40]. The combination of FMT and anti-PD-1 therapy was well-tolerated and conferred clinical benefits in 6 out of 15 patients [40]. Responders showed an increased abundance of taxa previously associated with the anti-PD-1 response, heightened activation of CD8+ T lymphocytes, and reduced frequency of myeloid cells expressing IL-8 [40].

Given these promising findings, several clinical trials are currently underway to explore the potential of FMT to enhance cancer therapy outcomes (Table 2).

**Table 2:** Summary of clinical trials evaluating the effects of FMT. Data were obtained from findings from www.clinicaltrials.gov using search terms “melanoma”, “Fecal microbiota transplant”, and “FMT”. Status of medical trials as of 27 September 2025

Treatment	Status	Enrolled patients	Primary outcome measures	NCT Number/ Reference
FMT, pembrolizumab	Phase 2 Completed	20	ORR^1^ [41]	NCT03341143
FMT, pembrolizumab, envatinib	Phase 2 Recruiting	56*	ORR^1^ [42]	NCT06030037
FMT, ICB^7^	Phase 1, Phase 2 Recruiting	24*	Efficacy, defined as clinical benefit SD^2^, PR^3^, CR^4^ [43]	NCT05251389
FMT, nivolumab	Phase 1 Unknown status	42*	Incidence of FMT^5^-related AE^6^, ORR^1^ [44]	NCT04521075

Note: ^1^Overall Response Rate per RECIST v1.1; ^2^Stable Disease; ^3^Partial Response; ^4^Complete Response; ^5^Fecal Microbiota Transplantation, ^6^Adverse Events; ^7^Immune Checkpoint Blockade; *Estimated.

### 3.3 Type A CpG (Cytosine-Phosphate-Guanine) TLR9 Agonist: Vidutolimod

TLR9 is a pattern recognition receptor predominantly expressed in dendritic cells, macrophages, NK cells, and other antigen-presenting cells [45]. Activation of TLR9 initiates an innate immune response that promotes the recognition and clearance of microbial pathogens and may contribute to the induction of tumor cell death [45].

Vidutolimod, previously referred to as CMP-001, is a viral mimic that stimulates the CpG-A receptor associated with TLR9 [45]. Its mechanism of action involves the induction of a type I IFN, leading to activation of T lymphocytes, which prompts an antitumor response [45]. This immunomodulatory effect has demonstrated potential in enhancing tumor sensitivity to PD-1 ICB [45].

Initial preclinical investigations indicated that TLR9 activators might serve as effective treatments, both alone and when paired with other therapeutic agents [45]. These findings led to the initiation of clinical trials involving patients with advanced malignancies. However, the observed clinical efficacy of TLR9 agonists to date has been limited [45]. Currently, numerous phase I and II clinical trials are exploring the application of TLR9 activators alongside chemotherapy, radiotherapy, targeted therapies, and immunotherapies (Table 3) [45].

**Table 3:** Summary of several ongoing Phases I and II clinical trials investigating TLR9 activators. Data were obtained from findings from www.clinicaltrials.gov using search terms “melanoma”, “CpG-A Toll Receptor 9”, and “anti-vidutolimod”. Status of medical trials as of 27 September 2025

Treatment	Status	Enrolled patients	Primary outcome measures	NCT Number/Reference
Vidutolimod, nivolumab	Phase 2 Completed	9	MPR^1^, pCR^2^, DMFS^3^ [46]	NCT04401995
CMP-001, nivolumab	Phase 2 Completed	34	MPR^1^ [47]	NCT03618641
Ipilimumab, tilsotolimod with ipilimumab	Phase 3, Terminated	481	Summary of independent reviewer-assessed ORR^4^, summary of overall survival^5^ [48]	NCT03445533

Note: ^1^Major Pathologic Response Rate; ^2^Pathologic Complete Response Rate; ^3^Distant-Metastasis Free Survival; ^4^Overall Response Rate per RECIST v1.1; ^5^Overall Survival.

In a study by Ribas et al., the combination of vidutolimod and pembrolizumab was evaluated. The results indicate that this treatment approach may be effective in addressing resistance to anti-PD-1 in approximately 25% of individuals suffering from metastatic melanoma, with manageable side effects [49].

The study conducted by Dongye et al. demonstrated comparable results, particularly with respect to the safety profile and the incidence of manageable adverse events. A quarter of the patients experienced a lasting therapeutic response, marked by tumor reduction in both local areas and distant locations, which included visceral metastases [50]. At first, individuals presenting noninflammatory tumor characteristics displayed significant responses to the treatment. However, post-therapy analysis revealed upregulation of the IFNγ gene signature [50].

### 3.4 Stimulator of the Interferon Gene (STING) Agonist via Natural Killer (NK) Cell Activation

The STING pathway is crucial for identifying cytosolic deoxyribonucleic acid (DNA) and cyclic dinucleotide (CDN) molecules, activating type I IFNs, and producing inflammatory cytokines essential for innate immunity [51]. This pathway is vital for cancer cell recognition. STING agonists, such as DNAs and CDNs, are expected to be potent adjuvants in cancer treatment because of their critical role in this process [51,52].

Recent findings have revealed that NK cells activated through stimulation of the STING pathway may serve as promising effectors in the anti-tumor response [52]. Consequently, the potential activation of NK cells by STING LNPs has been investigated to overcome tumor resistance to anti-PD-1 therapy [52]. A B16-F10 mouse melanoma model with lung metastasis, known for its resistance to anti-PD-1 therapy, was used in this study. Mice were intravenously administered STING-LNPs, and the mechanism underlying the enhancement of anti-PD-1 resistance by STING-LNPs was analyzed [52]. The combined application of STING-LNPs and anti-PD-1 demonstrated synergistic antitumor effects [52]. The results showed a significant increase in the expression of CD3 and CD4 lymphocytes, NK1.1, PD-1, and IFN-γ in lung metastases after STING-LNP treatment [52]. This observed change seems to be triggered by type I IFN generated by liver macrophages containing internalized STING-LNPs, leading to the systemic activation of PD-1-expressing NK cells. Activated NK cells, in turn, appear to produce IFN-γ, resulting in the upregulation of PD-L1 expression in cancer cells [52].

### 3.5 TBK1

INF regulatory factor 3 (IRF3) plays a role in the immune signaling pathway. Its activation and phosphorylation are mediated by two key kinases—TBK1 and IκB kinase epsilon (IKKε)—which are integral components of the STING [53]. These kinases are broadly expressed in lymphoid and myeloid immune cells. In addition to regulating IRF3 activity, they are involved in modulating the function of various immune cell populations, including T cells, B cells, dendritic cells, and macrophages. Their widespread expression and multifaceted roles in immune regulation present promising opportunities for therapeutic targeting in melanoma treatment [54].

In a study by Sun et al. [55], the molecular mechanisms underlying TBK1 activation and the effects of its inhibition were described. The findings suggest that TBK1 blockade may contribute to overcoming resistance to immunotherapy in melanoma by enhancing tumor cell sensitivity to immune-mediated responses [55]. Genetic deletion of TBK1 was shown to lower the cytotoxicity threshold of melanoma cells, thereby increasing their susceptibility to effector kinases and pro-inflammatory cytokines such as TNF-α and IFN-γ [55]. This modulation of the TME improves infiltration by cytotoxic CD8+ T cells and M1 macrophages, without inducing detrimental effects on overall immune function. Enhanced sensitization was observed when TBK1 inhibition was combined with ICB targeting PD-1 or its ligand [56].

Research on TBK1 and IKKε in human malignant melanoma remains in the preliminary stages. The therapeutic efficacy, safety profile, potential risks, and limitations of TBK1-targeted inhibition in the context of immunotherapy have not yet been fully elucidated [56–58]. Notably, TBK1 overexpression observed in melanoma harboring *NRAS* mutations presents additional complexity, as it may contribute to treatment resistance and reduced therapeutic effectiveness. Further investigation is required to clarify these mechanisms and to evaluate the potential benefits of combining TBK1 inhibition with established immunotherapeutic approaches [56–58].

### 3.6 LAG-3

*LAG-3* is a molecule found on the surface of immune cells, particularly T cells, where it acts as a negative regulator of their growth and activity [59]. In many tumors, including melanoma, *LAG-3* is expressed at higher levels, allowing tumors to dampen the immune response [59,60]. Therefore, blocking *LAG-3* has emerged as a promising therapeutic strategy [59–61]. Antibodies directed against *LAG-3* can enhance T-cell receptor (TCR) signaling and reduce the suppressive effects of regulatory T cells, thereby restoring a stronger antitumor response [59–61]. Importantly, *LAG-3* often works in conjunction with other immune checkpoints such as PD-1 and CTLA-4, and combined inhibition of these pathways offers significant potential to improve and expand the effectiveness of melanoma immunotherapy [59–61]. Relatlimab is the first human IgG4 *LAG-3*–blocking antibody that binds to *LAG-3* [61]. Its effectiveness was investigated in the RELATIVITY-047 trial, a global phase 2–3, double-blind, randomized study that compared a combination of relatlimab with nivolumab against nivolumab alone in patients with previously untreated metastatic or unresectable melanoma [61]. The results showed that the dual blockade of *LAG-3* and PD-1 was safe and the median progression-free survival was 10.1 months with relatlimab–nivolumab as compared with 4.6 months with nivolumab [61]. T-cell immunoglobulin and mucin-domain containing-3 (TIM-3) is another inhibitory receptor that modulates antitumor immunity [62]. It is expressed on exhausted CD4+ and CD8+ T cells, regulatory T cells, NK cells, and dendritic cells, where it interacts with ligands such as galectin-9, CEACAM-1, phosphatidylserine, and HMGB1 to suppress immune activation [62]. Acting in concert with LAG-3 and TIGIT, TIM-3 contributes to T-cell dysfunction within the tumour microenvironment, and its co-blockade with PD-1 has been shown to restore effector T-cell responses in cancer models [62].

### 3.7 TIGIT

TIGIT is a co-inhibitory receptor from the immunoglobulin superfamily [63]. Through several mechanisms, including the suppression of T-cell priming, it dampens the activity of both T cells and NK cells [63–65]. This results in a weakened anti-tumor immune response, making TIGIT an important target in cancer immunotherapy [63–65]. The interaction of TIGIT with its ligand CD155 delivers inhibitory signals directly to T lymphocytes and NK cells [63–65]. In cytotoxic T cells, this signaling promotes functional exhaustion, while in regulatory T cells it strengthens their immunosuppressive capacity [63–65]. Together, these effects contribute to the overall attenuation of the immune response [63–65]. Currently, anti-TIGIT monoclonal antibodies have been conducted in clinical trials in melanoma, however, the results of the study have not been reported [63–65].

### 3.8 OVs

The inception of oncolytic viral therapy (OVT) for cancer treatment dates back to the 1950s, rooted in the concept that viruses could infiltrate and eradicate cancer cells [66]. Early obstacles to this approach included challenges in achieving specificity towards cancer cells and dealing with toxicities [66]. However, subsequent discoveries revealed that OVT contributes to the elevation of cytokines such as INF-gamma and interleukins within the tumor microenvironment, which has the potential to enhance the innate immunologic response against tumor cells [66]. One prevalent approach to enhance the efficacy of OVs involves their combination with ICB [67]. This combined therapy proves effective in alleviating the immunosuppressive environment within tumors [67]. The infection induced by OVs serves as a catalyst for an anticancer immune response, thereby augmenting the impact of ICB [67]. Concurrently, ICB disrupt the ligand-receptor interactions of cancer cells, rendering them more susceptible to T cell attacks [67]. The primary aim of this combination strategy is to create a local microenvironment that is conducive to the optimal functionality of ICB, leveraging the infectious effects induced by OVs [67]. The FDA granted approval to Talimogene laherparepvec (T-VEC) in 2015 as the first OVT for treating unresectable melanoma [66,67]. T-VEC, an attenuated herpes simplex virus I (HSV-1), has been genetically modified to specifically target and eliminate cancer cells while preserving normal cells [66,67]. While T-VEC’s efficacy as a standalone treatment may have limitations, ongoing trials are exploring its immunostimulatory effects within the tumor microenvironment, particularly when used in combination with ICB [66,67]. Additionally, several other viruses are the subject of active clinical research as potential combined therapy with ICB [68,69]. In a pilot study, the combined therapy of ONCOS-102 (an oncolytic adenovirus expressing GM-CSF) with pembrolizumab was evaluated in patients with melanoma resistant to prior anti-PD-1 therapy [68]. Objective responses were observed in 7 out of 20 patients [68]. ONCOS-102 promoted the infiltration of T cells, especially cytotoxic CD8+ T cells, which persisted through the 9th week, providing clinical benefits [68]. In a phase 1b study, intratumoral therapy with the oncolytic virus V937 in combination with ipilimumab, an anti-CTLA-4 antibody, was evaluated in patients with advanced melanoma [69]. V937 is a bioselected, genetically unmodified Coxsackie A21 virus, which had previously demonstrated antitumor activity in patients with advanced melanoma. The objective response rate was 30% across all treated patients, 47% in those who hadn’t undergone prior anti-PD-1 therapy, and 21% in patients with disease progression during previous anti-PD-1 treatment [69].

## 4 Discussion and Conclusions

Melanoma is an aggressive form of skin cancer that requires timely and individualized therapeutic approaches to improve the patient’s outcomes [1,3]. Even after radical excision of the primary tumor, management of metastatic disease remains an important part of care [1–3]. Personalized treatment strategies are guided by the patient’s clinical and molecular profile [3]. Immunotherapy, in particular ICB, is associated with clinical benefit in the treatment of melanoma [10–12]. However, monotherapy with anti-PD-1 poses a risk of suboptimal therapeutic outcome, due to the rising number of acquired resistance against immunotherapy [12,20]. Therefore, there is a demand to explore and develop safe and effective combination therapies in order to provide a long-lasting, greater therapeutic benefit and to overcome resistance mechanisms [12,20].

One of the contributing factors to melanoma resistance to immunotherapy is immunosuppression within the TME, a feature typically absent in patients who respond to treatment [12,15]. Variability in therapeutic response may also result from tumor heterogeneity and resistance mechanisms, such as those mediated by lysine demethylase 5B (*KDM5B*) [20]. Further research is needed to identify predictive biomarkers and establish patient selection criteria for immunotherapy [12,13]. Combination approaches, such as TBK1 blockade and anti-PD-1, show therapeutic promise but require additional investigation to determine optimal combinatorial regimens and assess safety profiles [56–58]. Alternative strategies, including inhibition of T cell immunoreceptor with Ig and ITIM domains (TIGIT), protein arginine methyltransferase 7 (PRMT7), and the use of oncolytic viruses, may enhance the cytotoxic function of T lymphocytes and help overcome immunoresistance [63–65].

Synergistic treatment strategies combining immunotherapy with conventional therapies may enhance clinical outcomes by targeting distinct biological pathways [21–23]. However, further research and clinical development are required to optimize these approaches and reduce potential adverse effects. Variability in treatment response observed in large clinical trials highlights the genomic and epigenetic complexity of melanoma [18–20].

Preclinical studies have demonstrated that the combination of TBK1 inhibition with ICB results in an enhanced response to immunotherapy. Both TBK1 and TLR9 contribute to the upregulation of IFNγ signaling within the TME, thereby improving the efficacy of ICBs [45,55]. Clinical studies evaluating the combination of TLR9 agonists with anti-PD-1 have shown a favorable safety profile and durable responses, including tumor regression at both injected and non-injected sites [45].

Similarly, STING-LNPs also enhance IFNγ signaling, although through different mechanisms, primarily by activating NK cells and modulating CD3+ and CD4+ T-cell responses [52]. Additional studies are needed to assess the therapeutic efficacy and safety of this approach [52].

Another investigational strategy is the use of GM modulation. Data from patients with anti-PD-1-resistant melanoma suggest that FMT from treatment responders is well-tolerated and may provide clinical benefit [38–40]. Ongoing clinical trials are currently evaluating the combination of FMT with pembrolizumab or other therapies, highlighting the growing interest in microbiome-based approaches as adjuncts to cancer immunotherapy [38–40].

Targeted therapy using *BRAF* inhibitors, either as monotherapy or in combination with MEK inhibitors, modulates the complex immunological profile of the *BRAF* V600-mutant melanoma TME [18–20].

Clinical studies have shown that the addition of *BRAF*/MEK inhibitors to ICB in triplet combination regimens can improve PFS and enhance the durability of treatment responses [21–23]. However, the optimal integration and sequencing of triplet therapies in the first-line metastatic setting remains to be clearly defined. Ongoing clinical trials are evaluating the efficacy and safety of these therapeutic combinations to further refine the management strategies for patients with *BRAF* V600-mutant melanoma [21–23].

The goal of this review is to list promising new clinical possibilities and up-to-date studies still in early stages. We presented a wide array of potential therapeutic strategies. Some of them are ready for clinical practice (*BRAF* inhibitors, *BRAF*/MEK/ICB combined immunotherapy [18–20]), while others are still investigational (TBK1 [53,54], STING agonist [51,52], vidutolimod [45], FMT [38–40]).

We presented a variety of different clinical trials with diversified primary outcome measures, patient selection, enrollment size and predictive biomarkers. Consequently, reaching a definitive evaluation is difficult because we still need a lot more data about the potential clinical outcomes or possible irAE rates.

This review has several limitations. First, the available evidence is largely derived from early-phase clinical trials or preclinical studies, many of which included small patient cohorts and lacked long-term follow-up, limiting the strength and generalizability of conclusions. Second, the heterogeneity across study designs, endpoints, patient selection criteria, and outcome definitions makes direct comparison between studies difficult. Third, some of the examined therapeutic strategies, including TBK1 inhibitors, STING agonists, and FMT-based approaches, remain experimental, and the current evidence may not fully reflect their efficacy or safety in broader clinical practice. Finally, despite efforts to include recent and relevant studies, the review process may have missed unpublished data, negative results, or ongoing trials not yet indexed in major databases, which could influence the overall interpretation of emerging therapeutic trends.

## Supplementary Materials





## Data Availability

Not applicable.

## Supplementary Materials

The supplementary material is available online at https://www.techscience.com/doi/10.32604/or.2025.070505/s1.
